# Supplementary material for: Comparing hand-based and controller-based interactions in virtual reality learning: effects on presence and interaction performance
Source: PeerJ Comput Sci. 2025 Aug 28;11:e3168. doi: 10.7717/peerj-cs.3168 (PMC12453734; doi:10.7717/peerj-cs.3168)
Supplement: Supplemental Information 1 [file peerj-cs-11-3168-s001.pdf]

SURVEY FOR MEASURING THE FEELING OF PRESENCE IN VIRTUAL REALITY-BASED LEARNING ENVIRONMENTS

Hello dear students,

Below are some questions that will help us understand how you feel in learning environments using Virtual Reality (VR). We want you to answer these questions honestly and sincerely. Please do not leave any questions unanswered.

Check the most appropriate option for you as ☑.

What is your gender?

☐ Female ☐ Male

Have you ever used virtual reality for games or educational purposes?

☐ Yes ☐ No

How much were you able to control events?

☐ 1	☐ 2	☐ 3	☐ 4	☐ 5
Not compelling		Moderately compelling		Very compelling

How responsive was the environment to actions that you initiated (or performed)?

☐ 1	☐ 2	☐ 3	☐ 4	☐ 5
Not compelling		Moderately compelling		Very compelling

How natural did your hand/controller interactions with the environment seem?

☐ 1	☐ 2	☐ 3	☐ 4	☐ 5
Not compelling		Moderately compelling		Very compelling

How much did the visual aspects of the environment involve you?

☐ 1	☐ 2	☐ 3	☐ 4	☐ 5
Not compelling		Moderately compelling		Very compelling

How much did the auditory aspects of the environment involve you?

☐ 1	☐ 2	☐ 3	☐ 4	☐ 5
Not compelling		Moderately compelling		Very compelling

How natural was the mechanism which controlled movement through the environment?

☐ 1	☐ 2	☐ 3	☐ 4	☐ 5
Not compelling		Moderately compelling		Very compelling

How compelling was your sense of moving around inside the virtual environment?

☐ 1	☐ 2	☐ 3	☐ 4	☐ 5
Not compelling		Moderately compelling		Very compelling

How much did your experiences in the virtual environment seem consistent with your real world experiences?

☐ 1	☐ 2	☐ 3	☐ 4	☐ 5
Not compelling		Moderately compelling		Very compelling

Were you able to anticipate what would happen next in response to the actions that you performed?

☐ 1	☐ 2	☐ 3	☐ 4	☐ 5
Not compelling		Moderately compelling		Very compelling

How completely were you able to actively survey or search the environment using vision?

☐ 1	☐ 2	☐ 3	☐ 4	☐ 5
Not compelling		Moderately compelling		Very compelling

How well could you identify sounds?

☐ 1	☐ 2	☐ 3	☐ 4	☐ 5
Not compelling		Moderately compelling		Very compelling

How well could you localize sounds?

☐ 1	☐ 2	☐ 3	☐ 4	☐ 5
Not compelling		Moderately compelling		Very compelling

How well could you actively survey or search the virtu[illegible] environment using your hands/controller?

☐ 1	☐ 2	☐ 3	☐ 4	☐ 5
Not compelling		Moderately compelling		Very compelling

How compelling was your sense of moving around inside the virtual environment?

☐ 1	☐ 2	☐ 3	☐ 4	☐ 5
Not compelling		Moderately compelling		Very compelling

How closely were you able to examine objects?

☐ 1	☐ 2	☐ 3	☐ 4	☐ 5
Not compelling		Moderately compelling		Very compelling

How well could you examine objects from multiple viewpoints?

☐ 1	☐ 2	☐ 3	☐ 4	☐ 5
Not compelling		Moderately compelling		Very compelling

How involved were you in the virtual environment experience?

☐ 1 Not compelling | ☐ 2 | ☐ 3 Moderately compelling | ☐ 4 | ☐ 5 Very compelling

How much delay did you experience between your actions and expected outcomes?

☐ 1 Not compelling | ☐ 2 | ☐ 3 Moderately compelling | ☐ 4 | ☐ 5 Very compelling

How quickly did you adjust to the virtual environment experience?

☐ 1 Not compelling | ☐ 2 | ☐ 3 Moderately compelling | ☐ 4 | ☐ 5 Very compelling

How proficient in moving and interacting with the virtual environment did you feel at the end of the experience?

☐ 1 Not compelling | ☐ 2 | ☐ 3 Moderately compelling | ☐ 4 | ☐ 5 Very compelling

How much did the visual display quality interfere or distract you from performing assigned tasks or required activities?

☐ 1 Not compelling | ☐ 2 | ☐ 3 Moderately compelling | ☐ 4 | ☐ 5 Very compelling

How much did using your hands / controller interfere with the performance of assigned tasks or with other activities?

☐ 1 Not compelling | ☐ 2 | ☐ 3 Moderately compelling | ☐ 4 | ☐ 5 Very compelling

How well could you concentrate on the assigned tasks or required activities rather than on the mechanisms used to perform those tasks or activities?

☐ 1 Not compelling | ☐ 2 | ☐ 3 Moderately compelling | ☐ 4 | ☐ 5 Very compelling

How completely were your senses engaged in this experience?

☐ 1 Not compelling | ☐ 2 | ☐ 3 Moderately compelling | ☐ 4 | ☐ 5 Very compelling

How easy was it to identify objects through physical interaction; like touching an object, walking over a surface, or bumping into a wall or object?

☐ 1 Not compelling | ☐ 2 | ☐ 3 Moderately compelling | ☐ 4 | ☐ 5 Very compelling

Were there moments during the virtual environment experience when you felt completely focused on the task or environment?

☐ 1 Not compelling | ☐ 2 | ☐ 3 Moderately compelling | ☐ 4 | ☐ 5 Very compelling

How well could you move or manipulate objects in the virtual environment?

☐ 1 Not compelling | ☐ 2 | ☐ 3 Moderately compelling | ☐ 4 | ☐ 5 Very compelling

How easily did you adjust to using your hands / control devices used to interact with the virtual environment?

☐ 1 Not compelling | ☐ 2 | ☐ 3 Moderately compelling | ☐ 4 | ☐ 5 Very compelling

Was the information provided through different senses in the virtual environment (e.g., vision, hearing, touch) consistent?

☐ 1 Not compelling | ☐ 2 | ☐ 3 Moderately compelling | ☐ 4 | ☐ 5 Very compelling

The survey has ended. Thank you for your interest and contributions.
