# Supplementary material for: Comparing hand-based and controller-based interactions in virtual reality learning: effects on presence and interaction performance
Source: PeerJ Comput Sci. 2025 Aug 28;11:e3168. doi: 10.7717/peerj-cs.3168 (PMC12453734; doi:10.7717/peerj-cs.3168)
Supplement: Supplemental Information 2 [file peerj-cs-11-3168-s002.pdf]

SANAL GERÇEKLİK TEMELLİ ÖĞRENME ORTAMLARINDA BULUNUŞLUK HİSSİNİN ÖLÇÜLMESİ ANKETİ

Merhaba sevgili öğrenciler,

Aşağıda, Sanal Gerçeklik (SG) kullanılan öğrenme ortamlarında nasıl hissettiğinizi anlamamıza yardımcı olacak bazı sorular yer almaktadır. Bu soruları dürüst ve samimi bir şekilde cevaplamanızı istiyoruz. Lütfen hiçbir soruyu cevapsız bırakmayın.

Size göre en uygun seçeneği ☑ şeklinde işaretleyin.

Cinsiyetin?

☐ Kız ☐ Erkek

Hiç sanal gerçekliği oyun oynamak veya eğitim amaçlı kullandın mı?

☐ Evet ☐ Hayır

Olayları ne kadar kontrol edebildin?

☐ 1 ☐ 2 ☐ 3 ☐ 4 ☐ 5

Hiç Orta Tamamen

Başlattığın (veya gerçekleştirdiğin) eylemlere ortam ne kadar tepki verdi?

☐ 1 ☐ 2 ☐ 3 ☐ 4 ☐ 5

Hiç Orta Tamamen

Ortam ile etkileşimin ne kadar doğal görünüyordu?

☐ 1 ☐ 2 ☐ 3 ☐ 4 ☐ 5

Hiç Orta Tamamen

Ortamın görsel yönleri seni ne kadar içine aldı?

☐ 1 ☐ 2 ☐ 3 ☐ 4 ☐ 5

Hiç Orta Tamamen

Ortamdaki sesler seni ne kadar içine aldı?

☐ 1 ☐ 2 ☐ 3 ☐ 4 ☐ 5

Hiç Orta Tamamen

Ortam içerisinde hareketin kontrol edildiği sistem ne kadar doğaldı?

☐ 1 ☐ 2 ☐ 3 ☐ 4 ☐ 5

Hiç Orta Tamamen

Sanal ortam deneyimine ne kadar dâhil oldun?

☐ 1 ☐ 2 ☐ 3 ☐ 4 ☐ 5

Hiç Orta Tamamen

Ortamda hareket eden nesnelerin sende uyandırdığı his ne kadar inandırıcıydı?

☐ 1 ☐ 2 ☐ 3 ☐ 4 ☐ 5

Hiç Orta Tamamen

Sanal ortamdaki deneyimlerin, gerçek dünyadaki deneyimlerin ile ne kadar tutarlı görünüyordu?

☐ 1 ☐ 2 ☐ 3 ☐ 4 ☐ 5

Hiç Orta Tamamen

Gerçekleştirdiğin eylemlere karşılık olarak bir sonraki adımda ne olacağını tahmin edebildin mi?

☐ 1 ☐ 2 ☐ 3 ☐ 4 ☐ 5

Hiç Orta Tamamen

Gözlüğü kullanarak ortamı ne kadar aktif bir şekilde inceleyebildin veya araştırabildin?

☐ 1 ☐ 2 ☐ 3 ☐ 4 ☐ 5

Hiç Orta Tamamen

Sesleri ne kadar tanıyabildin?

☐ 1 ☐ 2 ☐ 3 ☐ 4 ☐ 5

Hiç Orta Tamamen

Seslerin geldiği yeri ne kadar belirleyebildin?

☐ 1 ☐ 2 ☐ 3 ☐ 4 ☐ 5

Hiç Orta Tamamen

Ellerini/kontrol kumandasını kullanarak sanal ortamı ne kadar aktif olarak inceleyebildin veya araştırabildin?

☐ 1 ☐ 2 ☐ 3 ☐ 4 ☐ 5

Hiç Orta Tamamen

Sanal ortam içerisindeki etrafta gezinme hissi ne kadar inandırıcıydı?

☐ 1 ☐ 2 ☐ 3 ☐ 4 ☐ 5

Hiç Orta Tamamen

Nesneleri ne kadar yakından inceleyebildin?

☐ 1 ☐ 2 ☐ 3 ☐ 4 ☐ 5

Hiç Orta Tamamen

Nesneleri farklı bakış açılarından ne kadar inceleyebildin?

☐ 1 ☐ 2 ☐ 3 ☐ 4 ☐ 5

Hiç Orta Tamamen

Sanal ortamdaki nesneleri ne kadar hareket ettirebildin veya yönlendirebildin?

☐ 1 ☐ 2 ☐ 3 ☐ 4 ☐ 5

Hiç Orta Tamamen

Ortamdaki hareketlerin ile hareketlerinin beklenen sonuçları arasında ne kadar gecikme yaşadın?

☐ 1 ☐ 2 ☐ 3 ☐ 4 ☐ 5

Hiç Orta Tamamen

Sanal ortam deneyimine ne kadar çabuk uyum sağladın?

☐ 1 ☐ 2 ☐ 3 ☐ 4 ☐ 5

Hiç Orta Tamamen

Yaşadığın deneyim sonrasında sanal ortamda hareket etme ve etkileşime girme konusunda kendini ne kadar yeterli hissettin?

☐ 1 ☐ 2 ☐ 3 ☐ 4 ☐ 5

Hiç Orta Tamamen

Verilen görevleri veya gerekli etkinlikleri yerine getirirken, gözlüğün görüntü kalitesi seni ne kadar engelledi veya dikkatini dağıttı?

☐ 1 ☐ 2 ☐ 3 ☐ 4 ☐ 5

Hiç Orta Tamamen

Kontrol cihazları, verilen görevlerin veya diğer etkinliklerin yerine getirilmesini ne kadar engelledi?

☐ 1 ☐ 2 ☐ 3 ☐ 4 ☐ 5

Hiç Orta Tamamen

Ortamdaki görev veya etkinlikleri yerine getirmek için kullanılan sistemlerden çok verilen görevlere veya etkinliklere ne kadar konsantre olabildin?

☐ 1 ☐ 2 ☐ 3 ☐ 4 ☐ 5

Hiç Orta Tamamen

Duyuların bu deneyimi ne kadar yoğun yaşadı?

☐ 1 ☐ 2 ☐ 3 ☐ 4 ☐ 5

Hiç Orta Tamamen

Nesneleri fiziksel etkileşim yoluyla tanımak (bir nesneye dokunmak, bir yüzeyin üzerinde yürümek veya bir duvar veya nesneye çarpmak) ne kadar kolay oldu?

☐ 1 ☐ 2 ☐ 3 ☐ 4 ☐ 5

Hiç Orta Tamamen

Sanal ortam deneyimi sırasında ortama veya göreve tamamen odaklandığını hissettiğin anlar oldu mu?

☐ 1 ☐ 2 ☐ 3 ☐ 4 ☐ 5

Hiç Orta Tamamen

Sanal ortamla etkileşim kurmak için ellerin/kontrol kumandasının kullanımına ne kadar kolay uyum sağladın?

☐ 1 ☐ 2 ☐ 3 ☐ 4 ☐ 5

Hiç Orta Tamamen

Sanal ortamda farklı duyularla sağlanan bilgiler (örneğin; görme, duyma, dokunma) tutarlı mıydı?

☐ 1 ☐ 2 ☐ 3 ☐ 4 ☐ 5

Hiç Orta Tamamen

Anket bitmiştir. İlginiz ve katkılarınız için teşekkür ederiz.
