# Supplementary material for: Comparing hand-based and controller-based interactions in virtual reality learning: effects on presence and interaction performance
Source: PeerJ Comput Sci. 2025 Aug 28;11:e3168. doi: 10.7717/peerj-cs.3168 (PMC12453734; doi:10.7717/peerj-cs.3168)
Supplement: Supplemental Information 3 [file peerj-cs-11-3168-s003.pdf]

Structured Observation Form

Observation Date: __________

Observer's Name: ________________________________

Student Name/ID: ________________________________

Experimental Group:

☐ Manual Interaction
☐ Controller-based Interaction

1. Technology Usage

1.1 Device Handling Skills

☐ Excellent (Uses the device with ease and without issues)
☐ Good (Generally uses the device correctly, rarely has issues)
☐ Fair (Experiences some difficulties in using the device)
☐ Poor (Experiences constant difficulties in using the device)

1.2 Following Instructions

☐ Excellent (Follows all instructions correctly and quickly)
☐ Good (Follows most instructions correctly)
☐ Fair (Has difficulty following some instructions)
☐ Poor (Fails to follow most instructions)

2. Interaction and Engagement

2.1 Interaction Level

☐ Very High (Constantly active and interactive)
☐ High (Mostly active and interactive)
☐ Medium (Occasionally active and interactive)
☐ Low (Rarely active and interactive)

2.2 Participation and Interest

☐ Very Interested (Shows high interest in all activities)
☐ Interested (Shows interest in most activities)
☐ Moderately Interested (Shows interest in some activities)
☐ Uninterested (Shows no interest in most activities)

3. Problem-Solving and Adaptability

3.1 Problem-Solving Skills

☐ Excellent (Solves encountered problems quickly and effectively)
☐ Good (Can solve most problems independently)
☐ Fair (Has difficulty solving some problems)
☐ Poor (Cannot solve most problems and constantly asks for help)

3.2 Adaptability

☐ Very High (Adapts quickly to changing situations)
☐ High (Adapts well to most situations)
☐ Medium (Has some difficulty adapting to certain situations)
☐ Low (Has difficulty adapting to most situations)

4. Overall Assessment

4.1 Overall Performance

☐ Excellent
☐ Good
☐ Fair
☐ Poor

4.2 Notes and Observations

____

____

____

____
